# Microwave Modification at Different Stages of Unsaturated Polyester/Brick Dust Composite Fabrication and Its Effect on Structural, Mechanical, Thermal and Moisture Properties

**DOI:** 10.3390/polym18131611

**Published:** 2026-06-28

**Authors:** Anton Mostovoy, Andrey Shcherbakov, Elvira Zhunussova, Ainur Duisenova, Amirbek Bekeshev

**Affiliations:** 1 Laboratory of Modern Methods of Research of Functional Materials and Systems, Yuri Gagarin State Technical University of Saratov, Polytechnichskaya St., 77, 410054 Saratov, Russia; 2 Laboratory of Support and Maintenance of the Educational Process, Yuri Gagarin State Technical University of Saratov, Polytechnichskaya St., 77, 410054 Saratov, Russia; gassmed7@gmail.com; 3 Department of Chemistry, Chemical Technology and Ecology, K. Kulazhanov Kazakh University of Technology and Business, Kayym Mukhamedkhanov St., Building 37 A, Astana 010000, Kazakhstan; tahmina.66@mail.ru; 4 Department of Physics, K. Zhubanov Aktobe Regional State University, Aliya Moldagulova Avenue 34, Aktobe 030000, Kazakhstan; ainura_duisenova@mail.ru; 5 Laboratory of Polymer Composites, K. Zhubanov Aktobe Regional State University, Aliya Moldagulova Avenue 34, Aktobe 030000, Kazakhstan

**Keywords:** unsaturated polyester resin, brick dust, technogenic waste, microwave post-curing, composite reinforcement, mechanical properties, thermal stability, water absorption, fracture morphology

## Abstract

The growing volume of industrial waste and the need for sustainable material solutions drive the search for cost-effective fillers and energy-efficient processing methods for polymer composites. This study investigates the valorization of brick dust (BD), a fine ceramic waste, as a reinforcing filler for unsaturated polyester resin (UPR), combined with microwave (MW) treatment applied at different stages of composite fabrication. The brick dust was comprehensively characterized using laser diffraction, SEM, EDX, XRD, and FTIR, revealing an environmentally safe aluminosilicate powder with a mean particle size of 3–6 µm, plate-like morphology, and surface hydroxyl groups favorable for matrix interaction. The optimal filler content was found to be 50 phr, which increased flexural strength by 6.5%, flexural modulus by 134%, tensile strength by 11%, and impact strength by 40% compared to neat UPR. Among the MW strategies evaluated, post-curing of the fully polymerized composite for 120 s proved most effective, yielding further improvements in flexural strength (110 MPa, +34.1%), flexural modulus (8250 MPa, +49.7%), impact strength (13.8 kJ/m^2^, +119%), and Shore D hardness (88). MW post-curing also increased the gel fraction from 95.0% to 97.8%, raised the thermal stability index (THRI) from 150.6 to 165.8, and reduced equilibrium water absorption from 0.62% to 0.47% with a reversibility index of 87.5%. Fracture surface analysis confirmed a transition from interfacial debonding to cohesive matrix failure, with ultra-thin polymeric veils replicating the scaly filler structure. These results demonstrate that microwave post-curing synergistically enhances the mechanical, thermal, and moisture-resistant properties of brick dust-filled polyester composites.

## 1. Introduction

The advancement of modern technologies and the tightening of environmental regulations are driving the search for novel approaches to the development of polymer composite materials with enhanced property profiles. In recent decades, there has been growing interest in the utilization of secondary resources and industrial waste as fillers for polymer matrices. This strategy not only reduces the cost of final products but also addresses the critical issue of industrial waste management, aligning with the principles of the circular economy and sustainable development [1,2].

Among the wide range of polymer matrices, unsaturated polyester resins (UPRs) occupy a prominent position. They are characterized by high processability, good physical and mechanical properties, and relatively low cost, which predetermines their extensive application as binders in construction materials, shipbuilding, automotive industry, and consumer goods manufacturing [3,4].

An analysis of the current scientific literature reveals that research on the modification of UPRs with technogenic waste is being conducted in several key directions. A significant body of work is devoted to the use of mineral fillers. For instance, Savotchenko and Kovaleva [3] investigated the effect of iron-containing fillers (iron ore concentrate, electrostatic precipitator dust) on the heat release kinetics and strength characteristics of PN-1 resin. The authors demonstrated that the introduction of coarse concentrate fractions accelerates curing, while the addition of organosilicon compounds increases the composite strength by 15–20%.

Another important direction involves the use of waste from natural stone processing and construction demolition. Baghloul et al. [5] established that incorporating 10–15 wt.% marble waste into UPR improves impact strength, tensile strength, and elastic modulus; however, higher filler content (20%) leads to embrittlement due to particle agglomeration. Nayak et al. [6] successfully applied marble dust as a secondary filler in glass-reinforced polyester composites, proving that its presence significantly enhances wear resistance. Bogiatzidis and Zoumpoulakis [7] demonstrated the feasibility of producing thermoset polymer composites with a high content (30–50 wt.%) of finely ground construction and demolition waste, achieving flexural strength values up to 60 MPa.

Other researchers have focused on optimizing the particle size distribution of fillers. Liu et al. [8] showed that optimizing the gradation of inorganic filler (CaCO_3_) dramatically increases the strength of highly filled UPR-based composites: average flexural strength increased by 343.5%, and compressive strength by 888.9%, due to reduced interfacial defects and an improved curing degree of the matrix.

The potential of agro-industrial waste is also being actively explored. Pączkowski et al. [9] studied composites based on UPR derived from recycled PET, filled with peanut shell powder, and evaluated their chemical resistance. Mahalingam et al. [10] used alkali-treated coconut tree secondary flower stalk fibers to fabricate composites suitable for sound and thermal insulation applications. Faria et al. [1] demonstrated that thermal modification of eucalyptus fibers (at 230 °C) reduces water absorption and enhances the strength of bio-based polyester composites.

Considerable attention is being paid to nanoscale and chemically modified fillers. Hsieh et al. [11] demonstrated that the incorporation of bio-derived chitin nanowhiskers into UPR significantly improves the adhesive properties of the material, with the dispersion medium playing a critical role. Embirsh et al. [2] investigated rice husk-derived biosilica modified with silanes and observed a substantial increase in hydrophobicity (up to 79.9%) and surface roughness of bio-based polyester nanocomposites. Kovačević et al. [12] studied the effect of acid-base treatment of non-metallic fractions from waste printed circuit boards on UPR properties, showing that the introduction of only 0.5 wt.% of treated filler increases tensile strength by up to 24.7% and reduces shrinkage.

The use of physical methods to influence the curing stage represents a promising avenue for enhancing the performance characteristics of polymer composites. In particular, microwave (MW) treatment can accelerate curing processes and affect the polymer structure. As demonstrated by Allayarov et al. [13], exposure to microwave and gamma radiation can significantly alter the deformation and strength properties of polyester resins, with the nature of these changes depending on the resin composition and processing conditions.

Despite the abundance of studies on the incorporation of various fillers, the use of such a common finely dispersed technogenic waste as brick dust—generated during brick production, construction, and demolition—as a filler in UPR matrices remains insufficiently explored. Brick dust, being an aluminosilicate material, has the potential to act as an active or inert filler, influencing the structure and properties of the composite. Comprehensive studies on the effect of microwave treatment on the structure and properties of brick dust-filled composites are absent from the available literature.

While previous studies have demonstrated the potential of various industrial wastes as fillers for unsaturated polyester resins and have explored microwave irradiation as a processing aid, the present study introduces several distinctive and previously unreported contributions. First, unlike most reports that focus on a single microwave treatment stage, this work systematically compares three different microwave application strategies—filler pretreatment, oligomer modification, and post-curing of the fully cured composite—within a single material system. Second, although brick dust has been occasionally mentioned as a potential filler, no comprehensive characterization of its phase composition, surface chemistry, and morphological evolution under microwave exposure has been reported. Third, this study provides direct experimental evidence for the formation of ultra-thin electron-transparent polymer veils replicating the lamellar structure of brick dust after microwave post-curing—a unique toughening mechanism not documented before for aluminosilicate-filled thermosets.

The central scientific question driving this study is whether microwave irradiation primarily affects the polymer matrix, the filler surface, or the interface, and whether the three treatment stages represent fundamentally different mechanisms or different manifestations of the same underlying phenomenon. While microwave treatment has been applied to polymers and composites, the mechanistic distinction between filler surface activation, matrix crosslinking acceleration, and post-curing network densification within a single material system has not been systematically addressed. Accordingly, the aim of the present work is to establish a unified mechanistic framework that distinguishes the dominant factors controlling each stage and clarifies how microwave interaction with polarizable species manifests differently depending on the stage of application, while simultaneously developing scientifically based approaches for the fabrication of high-performance UPR/BD composites.

## 2. Materials and Methods

### 2.1. Materials

#### 2.1.1. Polymer Matrix

The polymer matrix used in this study was an unsaturated polyester resin (UPR) based on orthophthalic acid, specifically Aropol™ M105TB (Ashland Inc., Wilmington, DE, USA). This resin is pre-accelerated and thixotropic, with a styrene content of approximately 37%. Its key technical characteristics are presented in Table 1. Methyl ethyl ketone peroxide (MEKP), commercially available as BUTANOX^®^ M-50 (Ashland Inc., USA), was used as the curing initiator. MEKP is a colorless transparent solution in dimethyl phthalate, with the properties listed in Table 2.

#### 2.1.2. Filler: Brick Dust

Brick dust (BD), a finely dispersed industrial waste generated during ceramic brick production, was used as the filler. The dust was collected from the filtration systems of JSC “Stroymaterialy. Engels Brick Plant” (Engels, Russia). The as-received dust was dried at 105 °C for 4 h prior to use to remove residual moisture [5].

Brick dust was selected as the filler over other construction and demolition wastes due to a combination of compositional, morphological, and practical advantages. Chemically, its aluminosilicate nature with surface hydroxyl groups enables hydrogen bonding with the polyester matrix, unlike inert fillers such as crushed concrete or glass powder. Morphologically, its inherent lamellar (plate-like) particle shape enhances mechanical interlocking and provides a tortuous barrier against crack propagation and moisture ingress. Critically, the brick dust used in this study is collected from the gas-cleaning filtration systems of ceramic brick manufacturing plants. This collection method ensures that the dust is already a finely dispersed powder with a consistently stable particle size (mean D50 = 3–6 μm) and does not require any additional milling, grinding, or classification prior to use. This is a substantial practical advantage over many other construction and demolition wastes (e.g., crushed concrete, brick aggregates, or roofing tile waste), which typically contain coarse fractions and require energy-intensive processing to achieve a suitable particle size distribution. Furthermore, brick dust from gas-cleaning filters is generated continuously as part of the manufacturing process, making it readily available at low cost with minimal variability. Finally, the absence of heavy metals and toxic elements (Section 3.1.1) ensures environmental safety, making brick dust particularly attractive for eco-composite applications where regulatory compliance is required.

### 2.2. Composite Preparation

#### 2.2.1. Filler Pretreatment

To enhance the interfacial interaction between the filler and the polymer matrix, two types of pretreatment were applied to the brick dust:

- Microwave activation: The brick dust was subjected to microwave irradiation at a frequency of 2.45 GHz and a power of 1.3 kW for 60 s using a laboratory microwave system. This treatment was intended to activate functional groups on the particle surfaces and improve subsequent dispersion [13].
- Ultrasonic dispersion: To reduce agglomeration, the filler was dispersed in the resin using an ultrasonic processor UZDN-2T (Russia) operating at 22 ± 2 kHz. The treatment was applied intermittently (2 min on/off cycles) for a total of 15 min. During sonication, the container was cooled with running water to prevent overheating of the resin.

#### 2.2.2. Curing Procedure

The composites were prepared by mechanically mixing the UPR with the brick dust at specified filler loadings (25, 50, 75 and 100 wt.%). After adding the initiator (2 wt.% MEKP), the mixture was degassed under vacuum to remove entrapped air. The curing process was conducted in two stages:

- Primary curing: 24 h at room temperature (25 °C);
- Post-curing: 8 h at 50 °C in a convection oven, followed by gradual cooling and relaxation for at least 24 h before testing [3].

For microwave-treated samples, all compositions were prepared with strictly identical mass and processed under the same fixed power (1.3 kW) and frequency (2.45 GHz). This ensures that energy input per unit mass was consistent across different treatment conditions, making the comparison between stages valid.

### 2.3. Characterization Methods

#### 2.3.1. Particle Size Analysis

The particle size distribution of the brick dust was determined by laser diffraction using a Fritsch Analysette-22 Nanotech (Fritsch GmbH, Idar-Oberstein, Germany), which covers a measurement range from 0.01 to 2000 µm. The analysis was performed in aqueous suspension, and the volume-based size distribution was calculated using the instrument’s software based on Mie scattering theory [8]. The particle size distribution was characterized by the following statistical parameters: D10 (10th percentile), D50 (median), and D90 (90th percentile), derived from the cumulative volume distribution. The reproducibility of the particle size distribution between different production batches was assessed by analyzing three independent samples collected from the same filtration system over a period of six months. Results are reported as mean ± standard deviation of three measurements per sample.

#### 2.3.2. Morphological Observation of Brick Dust

The morphology of the brick dust particles was examined by scanning electron microscopy (SEM) using a Tescan VEGA 3 SBH (Tescan, Brno, Czech Republic) operating at an accelerating voltage of 20 kV. Prior to observation, the powder samples were sputter-coated with a thin layer of gold to ensure electrical conductivity [7].

#### 2.3.3. Energy-Dispersive X-Ray Spectroscopy (EDX/EDS) Analysis

The elemental composition of the brick dust was analyzed by energy-dispersive X-ray spectroscopy (EDX/EDS) using an Oxford Instruments X-Max^5^ detector (UK) coupled with a Tescan VEGA 3 SBH scanning electron microscope (Czech Republic). The analysis was performed at an accelerating voltage of 20 kV with a working distance of 15 mm. Powder samples were mounted on carbon adhesive tabs and coated with a thin layer of gold to ensure electrical conductivity. Qualitative and quantitative analyses were performed by comparing the characteristic X-ray intensities of the elements in the sample with those in certified reference materials using the AZtec software platform (version 6.2). At least five different areas of each sample were analyzed to ensure representative results.

#### 2.3.4. X-Ray Diffraction (XRD) Analysis

The phase composition of the brick dust was investigated by X-ray diffraction (XRD) analysis. Diffraction patterns were recorded using an ARL X’tra diffractometer (Thermo Fisher Scientific, Tokyo, Japan) equipped with a CuKα radiation source (λ = 1.5406 Å). The measurements were performed in the 2θ angle range of 5–60° with a step size of 0.02° and a scan speed of 1°/min. The operating voltage and current were maintained at 40 kV and 40 mA, respectively.

Phase identification was carried out by comparing the obtained diffraction patterns with reference data from the International Centre for Diffraction Data (ICDD) Powder Diffraction File-2 (PDF-2) database using the Crystallographic Search-Match Program, version 3.1.0.2.b. The presence of an amorphous phase was inferred from the broad background hump observed in the diffraction pattern.

#### 2.3.5. Fourier Transform Infrared (FTIR) Spectroscopy of Brick Dust

The chemical structure and surface functional groups of the brick dust were investigated by FTIR spectroscopy using a Shimadzu IRTracer-100 (Shimadzu, Kyoto, Japan) spectrometer. Spectra were recorded in the range of 4000–400 cm^−1^ with a resolution of 4 cm^−1^, averaging 32 scans per sample. The samples were prepared as KBr pellets [4].

#### 2.3.6. Mechanical Testing

Mechanical properties of the composites were evaluated using an electromechanical universal testing machine WDW-5E (Time Group Inc., Beijing, China). All tests were conducted at room temperature (23 ± 2 °C) and 50 ± 5% relative humidity. At least five specimens were tested for each composition, and the results were averaged.

Tensile properties: Tensile strength (σ_t_) and tensile modulus of elasticity (E_t_) were determined according to ISO 527-1:2012 [14] at a crosshead speed of 2 mm/min using dumbbell-shaped specimens (type 1A).

Flexural properties: Flexural strength (σ_f_) and flexural modulus (E_f_) were measured according to ISO 178:2019 [15] using a three-point bending configuration with a support span of 60 mm and a testing speed of 2 mm/min. Specimen dimensions were 80 mm × 10 mm × 4 mm.

Impact strength: Charpy impact strength (a_i_) was determined on unnotched specimens according to ISO 179-1:2010 [16] using a pendulum impact tester LCT-50D (Beijing United Test Co., Beijing, China). Specimen dimensions were 80 mm × 10 mm × 4 mm [10].

#### 2.3.7. Thermal Analysis

Thermogravimetric analysis (TGA): Thermal stability and decomposition behavior were investigated using a MOM Q-1500 D derivatograph (Paulik-Paulik-Erdey, Hungarian Optical Works, Budapest, Hungary). Samples weighing approximately 100 mg were heated from 25 to 1000 °C at a rate of 10 °C/min under static air atmosphere. The relative error of mass change measurement did not exceed 1% [9]. The characteristic decomposition temperatures (T5%, T10%, T30%, T50%) were determined from thermogravimetric analysis (TGA) curves as the temperatures corresponding to 5%, 10%, 30%, and 50% mass loss, respectively [1,9]. The integral thermal stability index (THRI) was calculated using the following empirical equation, which combines information about the onset and progression of thermal decomposition [1,9]:

THRI = 0.49·[T_5%_ + 0.6·T_30_ − T_5%_]. (1)

This index provides a comprehensive measure of overall thermal stability, with higher values indicating greater thermal resistance.

Vicat softening temperature (VST): The Vicat softening temperature was determined according to ISO 306:2004 (Method B50) [17] using a load of 50 N and a heating rate of 50 °C/h.

#### 2.3.8. Water Absorption Measurements

Water absorption tests were conducted in accordance with ASTM D570-98 [18]. Specimens with dimensions of 60 mm × 60 mm × 4 mm were dried at 50 °C to constant mass (m_0_) and then immersed in distilled water at 23 ± 1 °C. At predetermined time intervals, specimens were removed, surface water was blotted with a lint-free cloth, and the mass (m_t_) was recorded using an analytical balance with an accuracy of ±0.1 mg. The water absorption at time t (W_t_) was calculated as:

(2) $$W_{t}=\frac{m_{t}-m_{0}}{m_{0}}\cdot 100\%$$

Measurements were continued until three consecutive weighings showed no significant change (<0.01%), indicating that equilibrium water absorption (W_∞_) had been achieved. Each reported value represents the mean of five specimens with standard deviations.

To assess the durability of composites after water immersion, the retention of mechanical properties was calculated as:

(3) $$Retention(\%)=\frac{P_{saturated}}{P_{dry}}\cdot 100\%$$

where P_dry_ is the property value for the dry sample and P_saturated_ is the property value after saturation.

The reversibility index (R_I_), which quantifies the proportion of degradation that is reversible upon drying, was calculated as:

(4) $$R_{I}=\frac{P_{re\text{-}dried}-P_{saturated}}{P_{dry}-P_{saturated}}\cdot 100\%$$

where P_re-dried_ is the property value after re-drying the saturated specimens at 60 °C for 24 h. This index ranges from 0% (completely irreversible degradation) to 100% (completely reversible degradation, i.e., pure plasticization).

The reversibility index is introduced here as a practical engineering metric to quantify the proportion of moisture-induced degradation that is reversible upon drying. Its conceptual foundation draws upon the well-established distinction in polymer composites between reversible plasticization (physical) and irreversible damage (chemical or mechanical) caused by water ingress [4,5,10,19,20]. While similar concepts have been used in other fields, this specific formulation is proposed here for the first time for evaluating moisture durability of polyester composites. The index provides a simple application-oriented measure of durability, as the ability of a composite to recover properties after drying is directly relevant to real-world service conditions involving cyclic wetting and drying.

#### 2.3.9. Calculation of Gel Fraction Content

The gel fraction content, representing the insoluble crosslinked portion of the polymer matrix, was determined by Soxhlet extraction according to ASTM D2765-01 [21]. Samples weighing approximately 1.0 g (m_0_) were extracted with refluxing acetone for 24 h. After extraction, the samples were dried at 60 °C to constant mass (m_1_). The gel fraction content (G) was calculated using the following equation:

(5) $$G=\frac{m_{1}}{m_{0}}\cdot 100\%$$

For filled composites, the mass of insoluble filler was taken into account by subtracting the known filler content from the initial sample mass. At least five specimens were tested for each composition, and the results were averaged.

## 3. Results

### 3.1. Characterization of Brick Dust as a Filler for Polymer Composites

The efficiency of particle-filled polymer composites is largely determined by the physicochemical properties of the dispersed filler, including its chemical composition, particle size distribution, morphology, and surface functionality [22]. In this study, brick dust (BD), an industrial waste generated during ceramic brick production, was thoroughly characterized prior to its incorporation into the unsaturated polyester resin matrix.

#### 3.1.1. Elemental Composition and Environmental Safety

**Figure 1:**
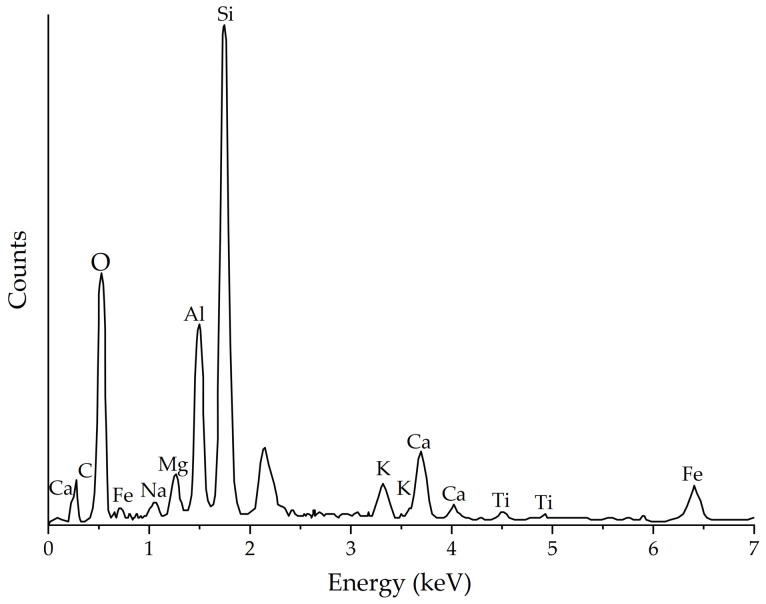
EDX spectrum of brick dust.

**Figure 2:**
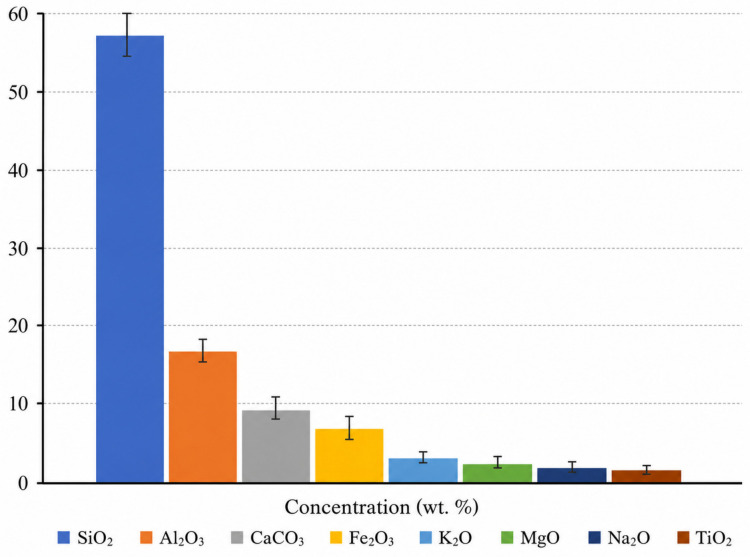
Quantitative results of X-ray fluorescence analysis of brick dust.

The elemental composition of the brick dust was determined by energy-dispersive X-ray spectroscopy (EDX/EDS) coupled with scanning electron microscopy. The obtained EDX spectrum (Figure 1) and the quantitative analysis results (Figure 2) reveal that the brick dust predominantly consists of silicon, aluminum, iron, magnesium, sodium, potassium, and titanium oxides, along with calcium carbonate. The chemical makeup of the ceramic filler is typical for clay-based materials and confirms its aluminosilicate nature.

As shown in Figure 2, silicon dioxide (SiO_2_) is the dominant phase at 55–60 wt.%, followed by aluminum oxide (Al_2_O_3_) at 15–18 wt.%. Recognizing the mineralogy of brick raw materials, calcium is presented as carbonate (CaCO_3_), calculated at 8–10 wt.%, indicating the use of a calcareous clay component. The material also contains iron oxide (Fe_2_O_3_, 6–8 wt.%), which acts as a flux and colorant, along with minor amounts of K_2_O, MgO, Na_2_O, and TiO_2_.

Notably, the EDX analysis did not detect any heavy metals or toxic elements above the permissible limits, confirming the environmental safety of the brick dust. This is a critical prerequisite for the development of eco-friendly composite materials intended for construction and consumer applications [23,24].

#### 3.1.2. Phase Composition by X-Ray Diffraction

**Figure 3:**
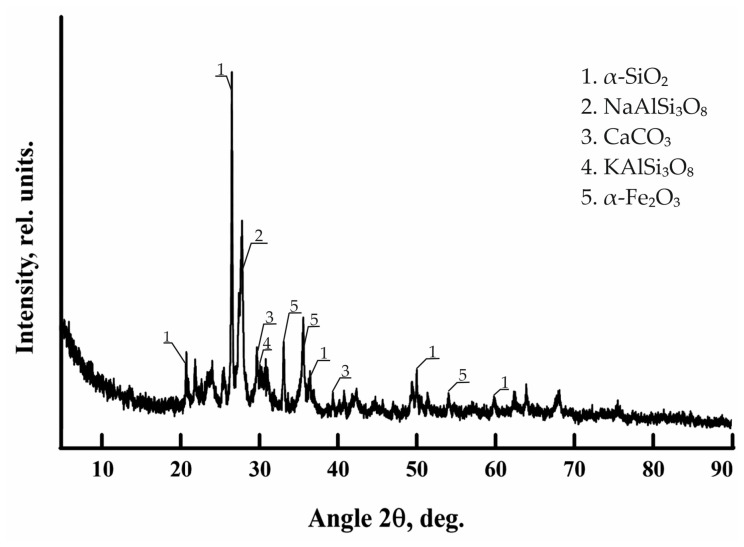
X-ray diffraction pattern of brick dust.

The phase composition of the brick dust was investigated by X-ray diffraction (XRD) analysis using a powder diffractometer with CuKα radiation (λ = 1.5406 Å) in the 2θ range of 10–90°. The obtained diffraction pattern (Figure 3) reveals a complex mixture of crystalline phases characteristic of clay-based ceramic materials.

The XRD pattern is dominated by intense reflections corresponding to quartz (α-SiO_2_), with the most intense peak at 26.6° 2θ. This confirms that quartz is the predominant crystalline phase, originating from the sand fraction in the raw clay mixture. The presence of hematite (α-Fe_2_O_3_) is confirmed by peaks at 33.2° and 35.6° 2θ. This phase is responsible for the characteristic reddish-brown color of ceramic bricks.

Feldspar minerals (albite, NaAlSi_3_O_8_, and microcline, KAlSi_3_O_8_) are identified by overlapping reflections in the 27–31° 2θ region, correlating with the presence of sodium, potassium, and aluminum oxides detected by EDX. A distinct peak at 29.4° 2θ suggests residual calcite (CaCO_3_), indicating the use of a calcareous clay component in the raw material. The broad hump in the background between 10 and 20° 2θ indicates the presence of an amorphous glassy phase, which forms during firing from the partial melting of feldspars and clay minerals [2,5,25]. This amorphous phase may contribute to enhanced interfacial interactions with the polymer matrix.

The phase composition revealed by XRD is fully consistent with the elemental composition determined by EDX analysis (Section 3.1.1) and confirms that brick dust is a complex aluminosilicate material with potential for active interaction with the polyester matrix.

#### 3.1.3. Fourier Transform Infrared Spectroscopy

**Figure 4:**
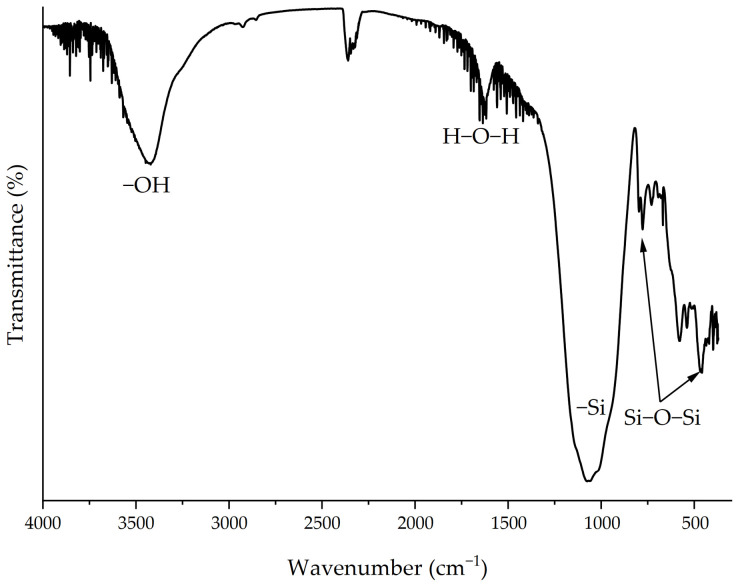
FTIR spectrum of brick dust.

The surface functional groups of the brick dust were investigated by FTIR spectroscopy. The FTIR spectrum (Figure 4) exhibits several characteristic absorption bands that provide insight into the chemical structure of the material.

The broad absorption band in the region of 3400–3450 cm^−1^ corresponds to O–H stretching vibrations of adsorbed water molecules and surface hydroxyl groups [26]. The band at 1630–1640 cm^−1^ is attributed to the bending vibrations of H–O–H, confirming the presence of molecular water. The intense broad band centered around 1030–1080 cm^−1^ is characteristic of asymmetric stretching vibrations of Si–O–Si and Si–O–Al bonds in aluminosilicates [26,27,28]. The bands at 780–800 cm^−1^ and 460–470 cm^−1^ correspond to symmetric stretching and bending vibrations of Si–O–Si bonds, respectively. Additionally, the weak band at 875 cm^−1^ and the band at 1420–1450 cm^−1^ indicate the presence of carbonate ions (CO_3_^2−^) from calcium carbonate [27,29].

The presence of surface hydroxyl groups and the hydrophilic nature of the aluminosilicate surface suggest the potential for favorable interactions with polar groups in the polyester matrix, which may enhance interfacial adhesion [30,31].

#### 3.1.4. Particle Size Distribution

**Figure 5:**
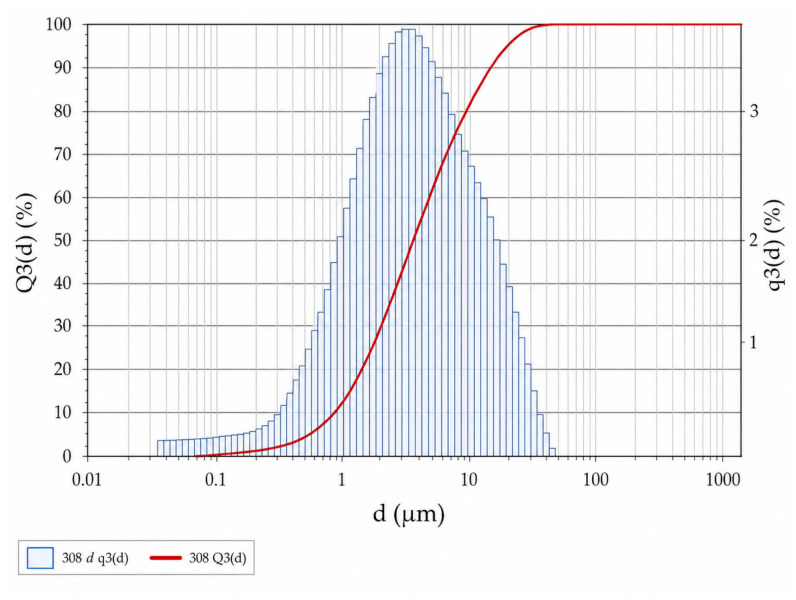
Particle size distribution of brick dust.

The particle size distribution of the brick dust was determined by laser diffraction analysis. The results (Figure 5) demonstrate a monomodal distribution with particle sizes ranging from 0.1 to 50 μm. The characteristic diameters, determined from the cumulative volume distribution, are as follows: D10 = 0.9 ± 0.1 μm, D50 = 4.0 ± 0.3 μm, and D90 = 13.2 ± 0.8 μm. The mean particle size (D50) was found to be in the range of 3–6 μm depending on the specific production batch, with a typical value of 4.0 μm for the batch used in this study. To assess batch-to-batch reproducibility, three independent samples collected from the same filtration system over a period of six months were analyzed. The D50 values varied by less than 8% (ranging from 3.7 to 4.4 μm), indicating good consistency of the brick dust generated by the gas-cleaning system of the brick manufacturing plant. Such a fine and stable particle size is advantageous for composite applications, as it promotes better packing density and provides a larger specific surface area for interaction with the polymer matrix [32,33]. The relatively narrow size distribution (span = (D90 − D10)/D50 ≈ 3.08) also suggests uniform filling capability, which is essential for maintaining consistent mechanical properties throughout the composite material [33,34].

#### 3.1.5. Morphological Characteristics

**Figure 6:**
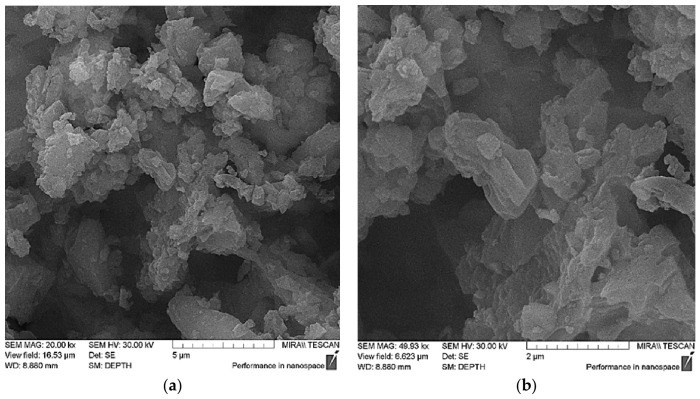
SEM micrographs of brick dust at different magnifications: (**a**) 20 kx; (**b**) 50 kx.

The morphology of the brick dust particles was examined by scanning electron microscopy (SEM). The SEM micrographs (Figure 6) reveal that the particles exhibit a predominantly plate-like structure with irregular shapes and angular edges.

The plate-like morphology is characteristic of clay-based materials after grinding and is associated with the layered structure of the original clay minerals. This morphology can be beneficial for composite reinforcement, as plate-like particles may contribute to improved barrier properties and mechanical interlocking with the polymer matrix. However, the irregular shape and angular edges may also lead to stress concentration points if not properly wetted by the resin [35,36,37].

#### 3.1.6. Summary and Implications for Composite Fabrication

The comprehensive characterization of brick dust reveals that this industrial waste possesses several features that make it a promising filler for unsaturated polyester composites:

- Chemical compatibility: the aluminosilicate composition with surface hydroxyl groups may enable hydrogen bonding and other interactions with the polyester matrix [26].
- Fine particle size: the mean particle size of 3–6 μm is suitable for achieving homogeneous dispersion and effective packing [38,39].
- Environmental safety: the absence of toxic elements confirms the suitability for sustainable material development [40,41].
- Plate-like morphology: the irregular, plate-like particles may contribute to mechanical interlocking and improved composite performance [35,42].

Based on these findings, brick dust was selected as a filler for the subsequent fabrication of UPR-based composites, with the expectation that it will act as an active filler capable of modifying the structure and properties of the polymer matrix. The following sections present the results of mechanical, thermal, and morphological investigations of the UPR/BD composites with varying filler content and under different processing conditions.

### 3.2. Physicomechanical Properties of UPR/Brick Dust Composites and Optimization of Filler Content

The incorporation of particulate fillers into polymer matrices is known to significantly alter the mechanical behavior of the resulting composites, with the extent and nature of these changes being highly dependent on the filler content, particle size, morphology, and interfacial interactions [43]. In this study, brick dust was incorporated into the unsaturated polyester resin (UPR) matrix at filler loadings ranging from 25 to 100 parts per hundred resin (phr). The resulting mechanical properties of the composites are summarized in Table 3.

#### 3.2.1. Physicomechanical Properties of UPR/Brick Dust Composites

The data presented in Table 3 reveal a non-monotonic dependence of the mechanical properties on the BD content, with the optimal performance observed at a filler loading of 50 phr.

Flexural Properties

As the BD content increased from 0 to 50 phr, the σ_f_ exhibited a gradual increase, reaching a maximum value of 82 MPa, which represents a 6.5% improvement compared to the unfilled resin (77 MPa). Concurrently, the E_f_ showed a dramatic increase from 2355 MPa for the neat UPR to 5510 MPa at 50 phr BD, corresponding to a 134% enhancement. This significant stiffening effect is attributed to the higher modulus of the inorganic filler particles compared to the polymer matrix, as well as to the effective stress transfer from the matrix to the rigid filler particles [39].

Further increase in BD content to 75 and 100 phr resulted in a slight decrease in flexural strength (to 80 and 78 MPa, respectively), while the flexural modulus continued to increase, reaching 9455 MPa at 100 phr—a 302% increase relative to the neat resin. This trend suggests that while the material becomes increasingly rigid at high filler loadings, the strength is compromised due to the onset of structural defects and insufficient matrix–filler interaction [44].

Tensile Properties

A similar pattern was observed for the tensile characteristics. The σ_t_ increased from 38 MPa for the neat UPR to 42 MPa at 50 phr BD (an 11% increase), then decreased to 38 MPa at 100 phr BD. The E_t_ increased progressively with filler content, from 1444 MPa (unfilled) to 4435 MPa at 100 phr BD—a 207% enhancement.

The increase in tensile modulus is a direct consequence of the incorporation of rigid inorganic particles that restrict the mobility of polymer chains and contribute to the overall stiffness of the composite [45]. The initial increase in tensile strength up to 50 phr indicates efficient stress transfer and good interfacial adhesion between the filler and the matrix. However, the decline in strength at higher loadings suggests that above an optimal filler concentration, the continuity of the polymer matrix is disrupted, and stress concentration points develop around filler agglomerates [45,46,47].

Impact Strength

The Charpy impact strength exhibited a pronounced maximum at 50 phr BD, increasing from 4.5 kJ/m^2^ for the neat resin to 6.3 kJ/m^2^—a 40% improvement. This enhancement indicates that the incorporation of brick dust at optimal loading increases the energy absorption capacity of the material during fracture. At lower filler contents (25 phr), the impact strength increased modestly to 4.8 kJ/m^2^, while at higher loadings (75 and 100 phr), it decreased to 4.6 and 3.4 kJ/m^2^, respectively, with the latter being lower than that of the unfilled resin.

Shore D hardness

In addition to the strength and modulus characteristics, the surface hardness of the composites was evaluated using the Shore D hardness test (Table 3). The neat UPR exhibits a hardness of 78 ± 2 Shore D, which is typical for unfilled polyester resins [4]. The incorporation of brick dust leads to a progressive increase in hardness, reaching 80 ± 2, 83 ± 2, 84 ± 2, and 85 ± 2 Shore D for composites containing 25, 50, 75, and 100 phr BD, respectively. This increase is attributed to the presence of rigid inorganic particles that resist local plastic deformation and indentation [5,6]. The most significant increment (from 78 to 83 Shore D) is observed upon the introduction of 50 phr BD, which correlates with the optimal filler loading identified for other mechanical properties. At higher filler contents (75 and 100 phr), the hardness continues to increase, but at a slower rate, reaching 85 Shore D at maximum loading. This trend suggests that even at high concentrations where strength may decline due to agglomeration, the surface layer retains high rigidity due to the high local concentration of hard particles [10]. The increased hardness of the composites, particularly at the optimal composition of 50 phr BD, makes them suitable for applications requiring enhanced surface durability and scratch resistance.

#### 3.2.2. Mechanisms of Reinforcement

The mechanical response observed in this study can be explained using the energy-based approach to composite reinforcement. Within this framework, the improvement in strength is associated with the greater amount of energy needed for fracture to occur. Several mechanisms contribute to this effect. One of them is the formation of additional fracture surface. As a crack moves through the composite, the dispersed filler particles obstruct its direct path, forcing it to propagate along a more complex route and thereby increasing the surface area generated during fracture. Another important factor is crack deflection and local bowing of the crack front. Rigid particles disturb the crack trajectory, causing deviations and increasing the effective crack length. This process raises the energy required for further crack growth. Energy is also dissipated through interfacial debonding and frictional interactions. Even when adhesion between the matrix and filler is favorable, local debonding may occur in the region ahead of the crack tip. This process consumes part of the applied energy, while subsequent frictional sliding between the matrix and the debonded particles provides an additional source of energy dissipation [45,48,49].

At the optimal filler loading of 50 phr, these mechanisms operate most effectively, resulting in the maximum observed strength and toughness. The decrease in strength at lower loadings (25 phr) can be attributed to an insufficient number of particles to effectively deflect cracks.

At higher filler concentrations (75–100 phr), the reduction in strength, particularly tensile and flexural strength, is a result of inefficient interaction between the polymer matrix and filler particles. At these loadings, the distance between particles becomes small enough that stress fields around adjacent particles overlap, creating regions of high local stress concentration [39,50]. Additionally, the probability of particle agglomeration increases, leading to the formation of structural defects that act as crack initiation sites [51,52,53].

From a molecular perspective, the presence of filler particles at optimal concentrations promotes the formation of a physical network in which adjacent BD particles are bridged by polymer macromolecules. This bridging effect restricts the conformational freedom of the polymer chains and limits their segmental mobility, resulting in increased modulus and strength. The formation of such “polymer bridges” between filler particles effectively transfers stress throughout the composite and contributes to the overall mechanical integrity [54,55].

The moderate improvements in strength compared to the substantial increases in modulus suggest that brick dust acts as a functional filler with moderate reinforcing efficiency rather than an inert extender. Its lamellar morphology promotes stiffening through mechanical interlocking and restricted chain mobility, while the transition to cohesive matrix failure (confirmed by SEM) and the significant increase in impact strength (+40%) indicate active energy dissipation. The modest strength gains reflect the inherent limitations of a technogenic ceramic filler with non-ideal morphology and surface defects, but the composite clearly exhibits reinforcing behavior rather than simple matrix dilution [39,45].

It is important to recognize that the 75 and 100 phr systems represent a fundamentally different structural regime compared to the 50 phr composite. Beyond the optimal loading, the material transitions from a matrix-dominated structure, where the polymer phase remains continuous and stress transfer is efficient, to a filler-dominated or agglomeration-controlled structure. At these high loadings, interparticle distances become sufficiently small that the system approaches critical packing density, leading to the formation of a percolating network of particles. The practical applicability of such highly filled systems is therefore limited to non-structural or lightly loaded components—such as acoustic panels, thermal insulation layers, or surface coatings—where high stiffness and hardness are advantageous and mechanical strength is less critical. For load-bearing applications, the 50 phr composition remains the optimal choice.

#### 3.2.3. Selection of Optimal Filler Content

Based on the comprehensive analysis of the mechanical properties, the optimal brick dust content for UPR-based composites is determined to be 50 phr, as at this concentration the composite exhibits maximum flexural strength (82 MPa, representing a 6.5% increase), significantly enhanced flexural modulus (5510 MPa, a 134% improvement), maximum tensile strength (42 MPa, an 11% increase), substantially increased tensile modulus (2875 MPa, a 99% enhancement), and peak impact strength (6.3 kJ/m^2^, a 40% increase compared to the unfilled resin). Furthermore, the Shore D hardness increases from 78 for the neat resin to 83 at 50 phr BD, representing a 6.4% improvement and indicating enhanced surface durability and resistance to local plastic deformation [4,5]. This combination of increased stiffness, retained strength, improved toughness, and enhanced hardness makes this composition particularly attractive for applications requiring balanced mechanical performance and surface durability.

We acknowledge that intermediate concentrations between 50 and 75 phr were not investigated; while the clear trend indicates the optimum lies near 50 phr, finer increments in this range could further refine the exact optimal loading.

These improvements indicate that at 50 phr, the brick dust acts as an effective reinforcing filler, creating a well-dispersed system with favorable matrix–filler interactions. The simultaneous enhancement of bulk mechanical properties and surface hardness confirms that the optimal filler loading provides benefits throughout the material structure, from the surface to the interior.

### 3.3. Effect of Microwave Modification at Different Stages of Composite Fabrication

Electrophysical methods of material processing have gained increasing attention as effective alternatives to traditional modification techniques for polymer composites. Among these, microwave (MW) irradiation offers distinct advantages, including reduced processing time, lower energy consumption, volumetric and inertia-free heating, improved environmental safety, and the ability to achieve precise temperature field distribution. In the context of polymer composites, microwave treatment can be applied at various stages of material preparation: pretreatment of the filler, modification of the uncured oligomer, or post-curing treatment of the fully polymerized composite [56,57].

In this study, microwave modification was investigated at three different stages of composite fabrication using the optimal composition identified in Section 3.2 (100 UPR: 50 BD):

1. MW pretreatment of brick dust (60 s at 2.45 GHz, 1.3 kW)—to activate the filler surface prior to incorporation into the resin;
2. MW modification of the uncured oligomer (3 s at 2.45 GHz, 1.3 kW)—immediately after mixing the resin with the filler and initiator;
3. MW post-curing of the fully polymerized composite (120 s at 2.45 GHz, 1.3 kW)—after complete curing of the material.

The mechanical properties of the composites obtained under these different treatment conditions were evaluated and compared with the untreated reference composite (100 UPR: 50 BD without MW modification). The results are summarized in Table 4.

#### 3.3.1. Microwave Pretreatment of Brick Dust

The application of microwave irradiation to the brick dust prior to its incorporation into the polymer matrix resulted in substantial improvements in the mechanical properties of the final composite, with the optimal effect observed at 60 s of treatment (Table 4). Compared to the untreated reference (100 UPR + 50 BD), the composite with BD irradiated for 60 s exhibited: flexural strength increased by 25.6% (from 82 to 103 MPa), flexural modulus increased by 30.6% (from 5510 to 7198 MPa), tensile strength increased by 35.7% (from 42 to 57 MPa), tensile modulus increased by 33.3% (from 2875 to 3833 MPa), and impact strength increased by 82.5% (from 6.3 to 11.5 kJ/m^2^). Shorter treatment (30 s) produced more modest improvements, while longer treatment (90 s) led to a slight decline in most properties, suggesting that 60 s is the optimal duration for surface activation without inducing thermal degradation of the filler particles.

These improvements may be attributed to the activation of the filler surface caused by microwave irradiation. At microwave frequencies, the electromagnetic field can induce molecular polarization and localized heating within the aluminosilicate particles. As a result, several surface-related changes may occur.

Microwave treatment may promote the desorption of physically adsorbed water and volatile impurities, thereby increasing the number of accessible active sites on the filler surface. It may also lead to the formation of additional surface hydroxyl groups, which can strengthen interfacial interactions with the polyester matrix through hydrogen bonding. In addition, partial decomposition of carbonate-containing phases may produce a more reactive surface. Changes in surface energy and wettability may further enhance resin penetration into surface irregularities, thereby improving matrix–filler adhesion [58,59,60].

The improved interfacial adhesion resulting from surface activation facilitates more efficient stress transfer from the matrix to the rigid filler particles, which is reflected in the enhanced strength and modulus values. The particularly pronounced increase in impact strength (82.5%) suggests that the treated particles interact more effectively with the matrix, promoting energy dissipation mechanisms such as particle debonding and matrix deformation rather than catastrophic crack propagation. This is also evidenced by the increase in Shore D hardness from 83 to 86 (Table 4), indicating that the surface layer benefits from the improved matrix–filler bonding, offering greater resistance to indentation and local plastic deformation [45,48,61].

#### 3.3.2. Microwave Modification of the Uncured Oligomer

Treatment of the uncured resin mixture immediately after incorporation of the filler and initiator was investigated at three different exposure times: 2, 3, and 5 s (Table 4). The optimal effect was observed at 3 s MW exposure, which led to significant improvements in mechanical properties compared to the untreated composite. Specifically, flexural strength increased by 9.8% (from 82 to 90 MPa), flexural modulus increased by 38.4% (from 5510 to 7625 MPa), tensile strength increased by 21.4% (from 42 to 51 MPa), tensile modulus increased by 16.5% (from 2875 to 3350 MPa), and impact strength increased by 66.7% (from 6.3 to 10.5 kJ/m^2^). Shorter treatment (2 s) produced only marginal improvements, with properties only slightly exceeding those of the untreated composite, while longer treatment (5 s) led to a decline in most properties compared to the 3 s treatment, suggesting that excessive exposure may initiate premature localized curing or induce thermal degradation of reactive components.

The relatively modest increase in flexural strength (9.8%) compared to the substantial increase in flexural modulus (38.4%) suggests that the primary effect of MW treatment at this stage is on the crosslinking density and network structure of the polymer matrix rather than on interfacial adhesion. Microwave irradiation of the uncured oligomer may accelerate initiator decomposition, thereby increasing the rate of free-radical generation and promoting polymerization at multiple sites simultaneously. Owing to the penetrating nature of microwave radiation, crosslinking may also proceed more uniformly throughout the bulk of the material. As a result, the formation of localized regions with low crosslinking density may be reduced. Microwave treatment may additionally influence the arrangement of polymer chains, potentially giving rise to a more uniformly crosslinked network [62,63,64].

The increase in modulus indicates the formation of a stiffer polymer network. At the same time, the improvement in strength and toughness suggests that more uniform crosslinking reduces the number of weak regions that can act as crack initiation sites. The slight increase in Shore D hardness from 83 to 85 (Table 4) further indicates that matrix modification alone contributes to surface hardening, although its effect is less pronounced than that of approaches aimed at improving interfacial adhesion, such as filler pretreatment or composite post-curing. Overall, the observed trend, namely a marked increase in modulus accompanied by more moderate improvements in strength and hardness, is typical of matrix-dominated property enhancement rather than interface-dominated reinforcement, which usually produces more balanced gains across the full set of mechanical properties [39,65].

#### 3.3.3. Microwave Post-Curing of Fully Polymerized Composites

The most substantial improvements in mechanical properties were observed when the fully cured composite was subjected to microwave post-treatment, with the optimal effect achieved at 120 s (Table 4). Compared to the untreated reference, the composite post-cured for 120 s exhibited: flexural strength increased by 34.1% (from 82 to 110 MPa), flexural modulus increased by 49.7% (from 5510 to 8250 MPa), tensile strength increased by 33.3% (from 42 to 56 MPa), tensile modulus increased by 18.6% (from 2875 to 3410 MPa), and impact strength increased by 119.0% (from 6.3 to 13.8 kJ/m^2^). Shorter treatment (60 s) produced limited improvements, while longer treatment (180 s) led to a decline in properties, particularly tensile strength and impact strength, indicating that excessive exposure may induce thermal degradation or microcracking.

The remarkable enhancement in mechanical properties, particularly the doubling of impact strength, indicates that microwave post-curing triggers multiple beneficial effects simultaneously. First, microwave irradiation of the already polymerized composite can induce additional crosslinking reactions by activating residual unsaturated groups or unreacted initiator fragments. Due to the volumetric nature of microwave heating, this post-curing process can occur more uniformly throughout the material, thereby increasing the overall crosslinking density and enhancing stiffness and strength [8,12]. Second, during conventional curing, differential shrinkage and thermal gradients can lead to the development of internal stresses within the composite, particularly at the matrix–filler interface, and microwave post-treatment, through selective heating of different phases, can promote molecular relaxation and stress relief, resulting in a more equilibrated structure with fewer microdefects [6,13]. Third, the differential heating of the filler particles and the polymer matrix under microwave irradiation may enhance interfacial bonding, as the aluminosilicate particles, with their higher dielectric loss, may heat more rapidly than the polymer, promoting localized annealing at the interface and potentially facilitating chemical interactions between surface functional groups and the matrix. Finally, the combination of thermal and electromagnetic effects may induce structural rearrangements within the polymer matrix, such as chain orientation or the formation of more ordered domains, which can contribute to improved mechanical performance [57,66].

The exceptional increase in impact strength (119%) suggests that the combination of improved interfacial adhesion, reduced internal stresses, and increased matrix toughness creates an optimal structure for energy dissipation during fracture, in which multiple energy-absorbing mechanisms operate synergistically: crack deflection by well-bonded particles, plastic deformation of the matrix, and controlled debonding at interfaces. The Shore D hardness reaches its maximum value of 88 (Table 4). This enhancement confirms that the surface layer benefits from the same structural improvements—increased crosslinking density, relief of internal stresses, and enhanced interfacial bonding—resulting in superior resistance to indentation and surface deformation [62,67]. The correlation between hardness and other mechanical properties further supports the conclusion that microwave post-curing creates a uniformly improved material structure throughout the bulk and at the surface.

#### 3.3.4. Comparative Analysis of Treatment Strategies

A comparative evaluation of the effectiveness of the three microwave treatment strategies, based on the percentage improvements in key mechanical properties relative to the untreated composite, is presented in Table 5.

From this comparison, several conclusions can be drawn:

1. Composite post-curing is the most effective overall treatment, yielding the highest improvements in flexural properties and impact strength. This approach addresses multiple aspects simultaneously: matrix crosslinking, interfacial adhesion, and stress relief.
2. Filler pretreatment is particularly effective for properties directly related to interfacial quality (tensile strength and modulus), confirming that surface activation enhances matrix–filler bonding.
3. Oligomer modification primarily affects matrix stiffness (flexural modulus) but yields more modest improvements in strength-related properties, suggesting that matrix modification alone is insufficient without concomitant improvement in interfacial adhesion.
4. The synergistic effects observed in the post-cured composite, particularly the dramatic increase in impact strength, indicate that the combination of enhanced matrix properties and improved interfacial characteristics is necessary for optimal toughness.

To confirm the proposed mechanisms of strengthening under microwave irradiation, the gel fraction content of the samples after extraction in acetone was investigated (Table 6).

The control sample without MW treatment is characterized by a gel fraction content of 95.0%, which corresponds to the typical degree of curing for this system [2,7]. Preliminary microwave treatment of brick dust (60 s) increases this value to 95.8%, which is associated with the activation of the filler surface and improved adsorption of polymer chains; however, the matrix itself does not undergo additional crosslinking [68]. Treatment of the uncured oligomer (3 s) provides a more substantial increase in gel fraction to 96.5% due to accelerated and uniform initiation of crosslinking reactions throughout the material volume [56,69]. The maximum gel fraction value (97.8%) was achieved by post-curing the already formed composite for 120 s, indicating the occurrence of additional reactions involving residual unsaturated groups and the relaxation of internal stresses, which contributes to the formation of a more perfect three-dimensional network [70,71]. Thus, the gel fraction data fully confirm that microwave treatment, especially at the post-curing stage, leads to an increase in the crosslinking density of the polyester matrix, which in turn determines the observed improvement in the mechanical characteristics.

Synthesizing the findings from the three treatment stages, a unified mechanistic model can be proposed. The three stages operate through the same fundamental physical phenomenon—the interaction of microwave radiation with polarizable species (dipolar molecules and polarizable surfaces)—but manifest differently depending on which component is exposed. Filler pretreatment primarily activates the filler surface through desorption of moisture and generation of hydroxyl groups, enhancing interfacial adhesion as the dominant mechanism. Oligomer modification primarily affects the polymer matrix by accelerating initiator decomposition and promoting more uniform crosslinking. Composite post-curing combines both effects but operates primarily through additional crosslinking of the matrix, complemented by stress relief and interfacial enhancement. This framework provides a clear theoretical basis for understanding the observed differences in property improvements across the three stages, with the dominant factor shifting from interfacial chemistry (filler pretreatment) to matrix crosslinking kinetics (oligomer modification) to network densification and stress relaxation (composite post-curing).

Based on the comprehensive analysis of the mechanical properties, microwave post-curing of the fully polymerized composite for 120 s was identified as the most effective treatment strategy for UPR/BD composites at the optimal filler loading of 50 phr. This treatment resulted in the highest flexural strength of 110 MPa, corresponding to a 34.1% increase, and the highest flexural modulus of 8250 MPa, representing a 49.7% improvement. It also produced a high tensile strength of 56 MPa, a 33.3% increase, and a substantial increase in tensile modulus to 3410 MPa, corresponding to an 18.6% improvement. In addition, the impact strength reached 13.8 kJ/m^2^, which represents an exceptional 119.0% increase compared with the untreated composite. The pronounced improvement in impact toughness, together with significant gains in strength and stiffness, makes this treatment particularly promising for applications requiring high-performance materials with a balanced combination of mechanical properties.

### 3.4. Morphological Analysis of Fracture Surfaces

Scanning electron microscopy analysis of the fracture surfaces reveals a pronounced evolution of morphology depending on the microwave treatment strategy. The observed microstructural effects provide direct evidence for the underlying physicochemical mechanisms governing the mechanical performance of the composites.

#### 3.4.1. Fracture Surface Morphology of Neat UPR

**Figure 7:**
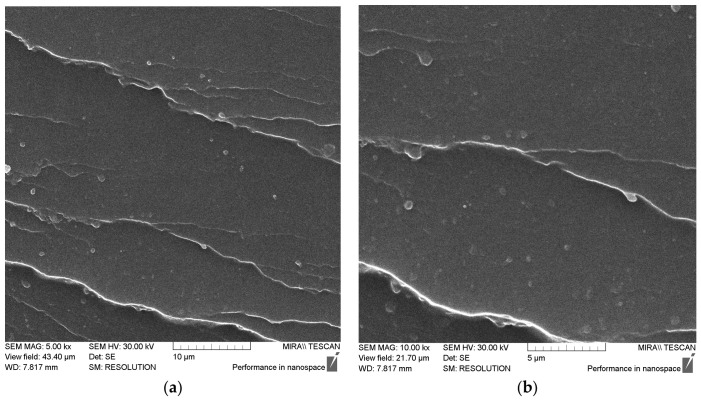
SEM micrographs of neat UPR at different magnifications: (**a**) 5 kx; (**b**) 10 kx.

The fracture surface of the unmodified polyester resin exhibits classical brittle cohesive fracture typical of unsaturated polyester resins. The surface is relatively smooth with well-defined fracture facets and distinct crack propagation lines, indicating low energy dissipation during fracture (Figure 7) [72,73].

#### 3.4.2. Fracture Surface Morphology of the UPR Composite Filled with Brick Dust

**Figure 8:**
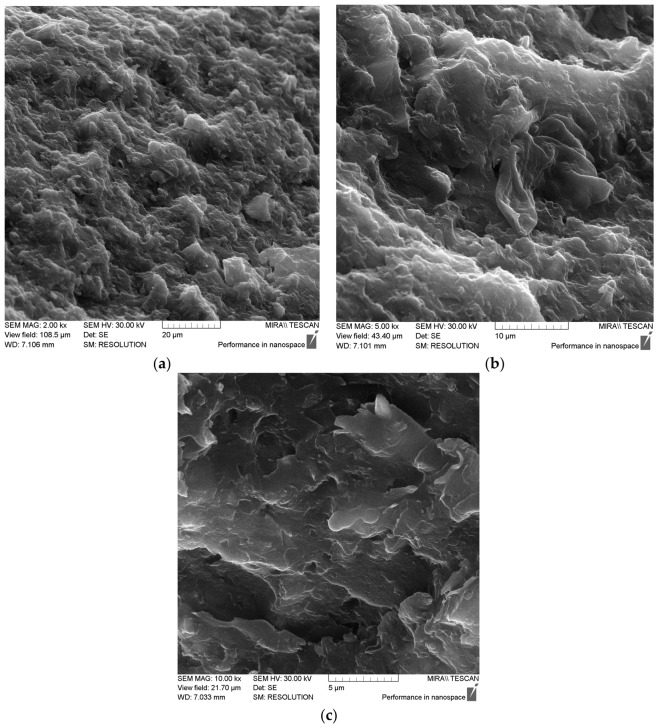
SEM micrographs of brick-dust-filled UPR composites at different magnifications: (**a**) 2 kx; (**b**) 5 kx; (**c**) 10 kx.

The incorporation of brick dust alters the fracture behavior, resulting in a more heterogeneous fracture mode. The fracture surface becomes noticeably rougher, and discrete mineral filler particles are clearly visible. Although the matrix–filler adhesion appears generally satisfactory, local regions of incomplete contact between the matrix and the dispersed phase can still be observed, which is typical of composites prepared by mechanical mixing without additional surface activation (Figure 8) [73,74].

#### 3.4.3. Fracture Surface Morphology of the UPR Composite Filled with Microwave-Treated Brick Dust

**Figure 9:**
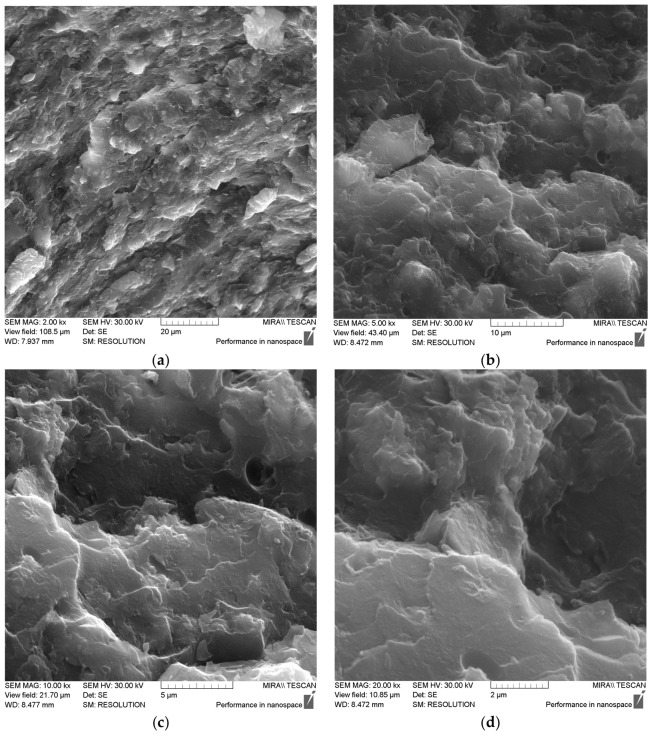
SEM micrographs of the UPR composite filled with microwave-treated brick dust at different magnifications: (**a**) 2 kx; (**b**) 5 kx; (**c**) 10 kx; (**d**) 20 kx.

Preliminary microwave treatment of the brick dust leads to a visually more uniform distribution of particles within the matrix. The fracture surface exhibits a finer microrelief compared to the untreated composition, indicating improved interfacial contact (Figure 9).

#### 3.4.4. Fracture Surface Morphology of the UPR Composite Prepared from Microwave-Treated BD-Filled Oligomer

**Figure 10:**
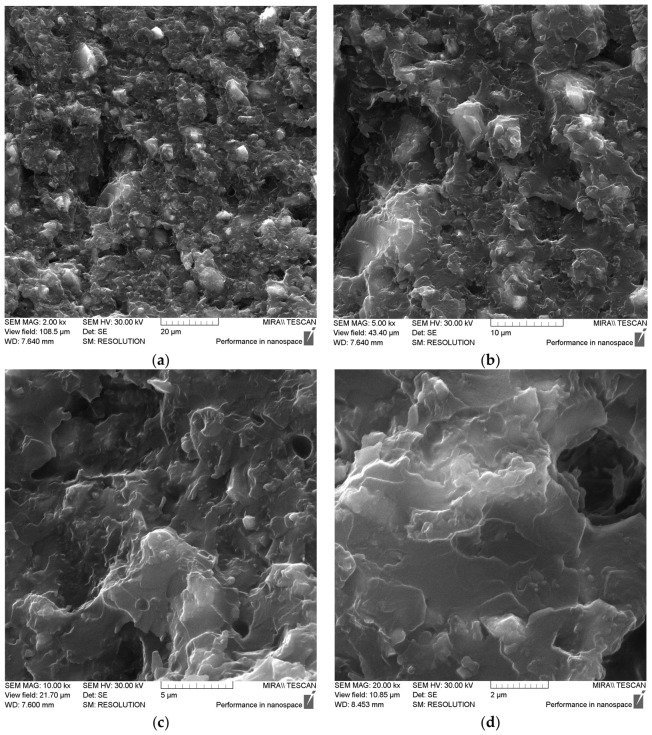
SEM micrographs of the UPR composite prepared from microwave-treated BD-filled oligomer at different magnifications: (**a**) 2 kx; (**b**) 5 kx; (**c**) 10 kx; (**d**) 20 kx.

Microwave exposure of the uncured polyester resin results in the formation of a pronounced scaly or ductile fracture structure (Figure 10). The resin more tightly encapsulates the agglomerates, and the matrix appears more structured, indicating a change in the crack propagation path [62,64].

#### 3.4.5. Fracture Surface Morphology of the UPR-BD Composite After Microwave Post-Treatment

**Figure 11:**
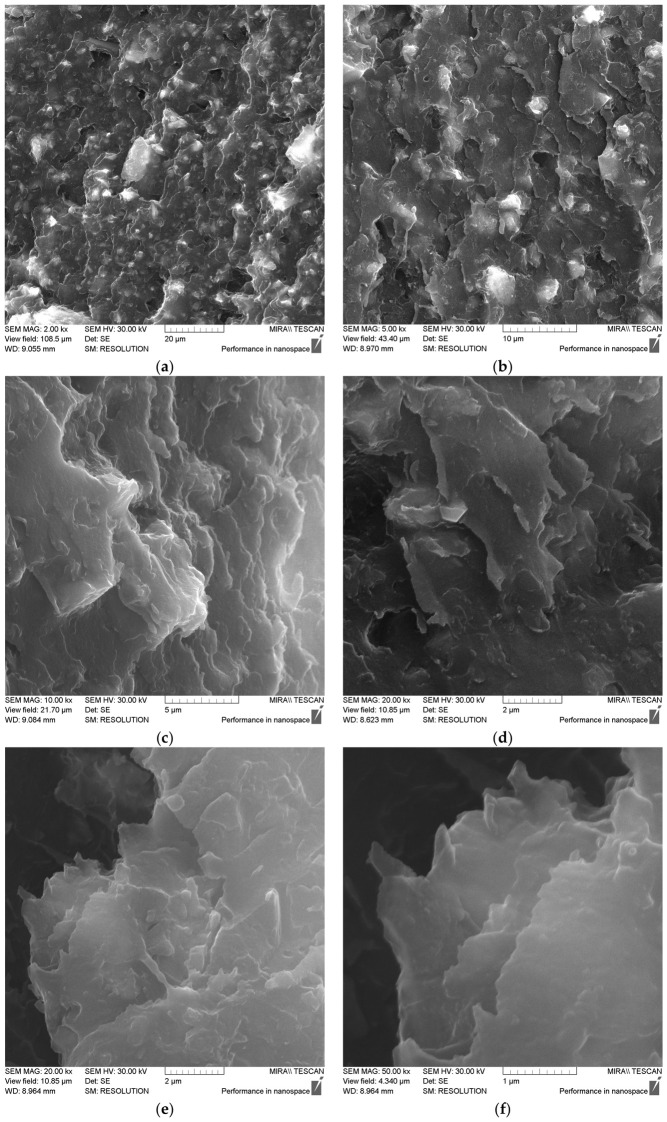
SEM micrographs of the UPR-BD composite after microwave post-treatment at different magnifications: (**a**) 2 kx; (**b**) 5 kx; (**c**) 10 kx; (**d**) 20 kx; (**e**) 20 kx; (**f**) 50 kx.

This treatment strategy yields the most complex and tortuous fracture surface topology. Excellent encapsulation of brick dust particles by the polymer matrix is observed, with virtually no visible interfacial debonding or particle pull-outs (Figure 11). This indicates a transition from adhesive failure (at the phase interface) to cohesive failure (within the matrix bulk) [75,76,77].

#### 3.4.6. High-Resolution Observations and Proposed Mechanisms

The observed microstructural transformations can be explained by several factors arising from the specific nature of dipolar polarization under microwave field exposure. Brick dust, as a mineral aluminosilicate material, tends to adsorb moisture. Microwave treatment of the filler induces intense volumetric heating and deep desorption of bound water from micropores. This liberates active sites (e.g., hydroxyl groups) on the particle surface, significantly improving the wettability of the hydrophilic filler by the hydrophobic polyester matrix and reducing the interfacial surface tension [78]. Additionally, microwave radiation affects the polar ester groups of the oligomer, temporarily but dramatically reducing the system viscosity and allowing the filler to distribute much more homogeneously. The microwave field also initiates local hot-spots that serve as centers for accelerated polymerization [79,80]. Furthermore, the superior polymer–filler adhesion in the microwave post-cured composite is explained by a synergistic effect, whereby simultaneous internal heating of both the reactive mass and the brick dust particles ensures maximum curing depth. Such a tortuous fracture morphology directly correlates with the formation of a dense three-dimensional crosslinked network [79].

#### 3.4.7. Ultra-Thin Polymeric Veils and Electron Transparency

A particularly noteworthy effect is observed at higher magnifications (×20,000). The visual effect of “electron transparency” at a relatively high accelerating voltage (30 kV) provides direct evidence that the thickness of these plate-like structures is extremely small—on the order of tens or low hundreds of nanometers.

The formation of such ultra-thin structures on the fractograph surface can be explained by several physicochemical mechanisms. Crosslinked polyester resins are classified as brittle matrices. However, the introduction of dispersed particles combined with microwave treatment dramatically alters the stress distribution at the tip of the propagating main crack. During sample fracture, high shear stresses develop in the zones adjacent to the filler particles. The matrix may locally transition into a forced elastic state, drawing out into ultrathin films (craze-like structures). Upon complete crack opening, these overstressed microfilms rupture and deposit on the fracture surface as translucent polymer scales [48].

Microwave treatment ensures exceptionally strong adhesion between the liquid oligomer and the solid phase. Consequently, a boundary layer of polymer with altered network conformation and increased density develops around each mineral particle. In this case, the adhesive bond strength exceeds the cohesive strength of the matrix. As a result, failure occurs cohesively, but not within the bulk of the neat resin. Rather, it involves the detachment of thin fragments of the restructured boundary layer, which reflect the flat crystalline geometry of the filler [39,79,81].

#### 3.4.8. Bright Edge Features and Fracture Energy Dissipation

At higher magnifications (×50,000), clusters of bright, “luminous” regions are observed at the fracture edges. Since the polyester surface was sputter-coated with gold prior to SEM examination, charge accumulation on the non-conductive polymer was largely suppressed, and the observed bright contrast is therefore more reasonably attributed to topographic contrast formation in secondary-electron imaging rather than to severe charging artifacts. In secondary-electron mode, sharp edges, protrusions, and torn thin regions appear especially bright because of the edge effect and enhanced local secondary-electron emission. Thus, these “white particles” are more likely microscopic peaks, jagged ridges, and elongated fragments of the polyester matrix itself, rather than detached debris or foreign inclusions [82].

In rigid thermosets, the appearance of elongated, irregular fracture ridges suggests that localized energy-dissipating deformation preceded final crack advance. Instead of propagating through a smooth and relatively unperturbed brittle path, the crack interacted with the dispersed phase, which promoted localized plastic deformation, crack deflection, and crack pinning. In such regions, the matrix may be drawn into craze-like fibrillar ligaments before final rupture occurs [83,84].

This morphology, characterized by abundant jagged edges, is consistent with strong polymer–filler interfacial interaction and enhanced fracture-energy dissipation. Microwave post-curing promotes deeper and more uniform curing through volumetric heating of the composite, which can improve interfacial integrity and reduce the tendency to interfacial debonding. As a result, fracture proceeds predominantly by cohesive failure within the matrix or interphase-adjacent regions rather than by particle–matrix separation. The increased density of structural bonds requires higher energy for crack propagation, which is dissipated through crack deflection, localized deformation, and the formation of a complex new fracture surface manifested as jagged tear edges [79,83,85].

#### 3.4.9. Summary of Morphological Findings

The SEM fractography presented in this study provides multiple lines of direct evidence for enhanced interfacial adhesion in the microwave post-cured composite, which collectively allow us to attribute the observed property improvements to improved matrix–filler bonding without relying on indirect inference. First, the complete absence of particle pull-out cavities in the MW post-cured samples (Figure 11) versus their abundance in the untreated composite (Figure 8) directly demonstrates that the interfacial bond strength exceeds the cohesive strength of the matrix, such that fracture propagates through the polymer rather than along the interface [75,76]. Second, the observation of thin polymer layers remaining firmly attached to the exposed filler surfaces after fracture, with no visible gaps or dewetting, provides direct visual evidence of strong adhesion at the molecular scale [39,79]. Third, the formation of ultra-thin electron-transparent polymer veils replicating the lamellar morphology of the brick dust particles (Section 3.4.7) indicates that the polymer matrix was able to intimately wet and conform to the filler surface topography prior to fracture, which is a hallmark of superior interfacial contact [48]. Fourth, the transition from a relatively smooth fracture surface in the untreated composite to a rough, tortuous morphology with jagged tear edges and extensive crack deflection in the MW post-cured composite is characteristic of cohesive failure within the matrix rather than adhesive failure at the interface, providing additional visual evidence of strong bonding [83,84]. Fifth, the progressively increasing gel fraction content (from 95.0% to 97.8%, Table 6) indirectly supports the morphological observations, as higher crosslinking density enhances the cohesive strength of the matrix and reduces the tendency for interfacial failure [56,69]. Collectively, these complementary observations, derived from high-resolution SEM fractography and supported by gel fraction measurements, constitute robust qualitative and semi-quantitative evidence for improved interfacial adhesion in the microwave post-cured composite.

### 3.5. Thermal Properties

**Figure 12:**
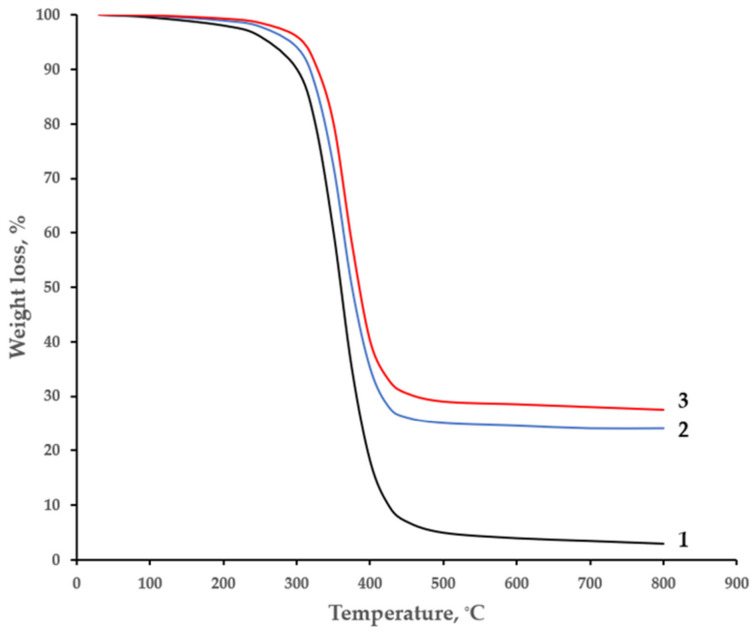
TGA curves of UPR/BD compositions: 1—Neat UPR; 2—UPR + 50 BD; 3—UPR + 50 BD + MW Post-cure.

The thermal stability of polymer composites is a critical parameter governing their applicability in elevated temperature environments and providing insight into the structural characteristics of the material, particularly the crosslinking density and the quality of matrix–filler interfacial interactions [86]. In this study, the thermal behavior of the unsaturated polyester resin composites filled with brick dust was investigated by thermogravimetric analysis (TGA), (Figure 12) and Vicat softening temperature measurements. Three representative samples were analyzed: neat UPR (unfilled resin), the optimal composite formulation with 50 phr BD without microwave treatment, and the same formulation subjected to microwave post-curing for 120 s (identified as the optimal treatment in Section 3.3). The thermal stability parameters derived from TGA data, including characteristic decomposition temperatures and the integral thermal stability index (THRI), are summarized in Table 7, together with the Vicat softening temperatures (T_v_).

The introduction of brick dust into the UPR matrix significantly enhances the thermal stability of the resulting composite. Compared to the neat resin, the composite containing 50 phr BD exhibits substantially higher decomposition temperatures across all characteristic points. The temperature of 5% mass loss increases from 262 °C for the unfilled resin to 286 °C for the filled composite, representing a 24 °C improvement. Similarly, T_10%_ increases by 15 °C (from 300 to 315 °C), T_30%_ increases by 15 °C (from 337 to 352 °C), and T_50%_ increases by 19 °C (from 365 to 384 °C). These shifts toward higher temperatures indicate that the presence of brick dust particles effectively delays the thermal degradation of the polyester matrix. Several mechanisms may contribute to the improved thermal stability. One of them is the physical barrier effect, in which dispersed inorganic particles hinder the diffusion of volatile decomposition products from the bulk material to the surface. Another factor is the restricted mobility of polymer chains caused by their interactions with the filler surface, which increases the energy required for chain scission. In addition, more uniform heat dissipation within the composite may reduce localized overheating and thereby slow down thermal degradation [87,88,89].

Microwave post-curing of the fully polymerized composite for 120 s results in further improvement of thermal stability. The MW-treated composite exhibits T_5%_ = 308 °C, which is 22 °C higher than the untreated filled composite and 46 °C higher than the neat resin. Similar enhancements are observed for T_10%_ (335 °C, +20 °C relative to untreated filled composite), T_30%_ (360 °C, +8 °C), and T_50%_ (390 °C, +6 °C). The enhanced thermal stability of the microwave-treated composite can be attributed to the microstructural changes induced by microwave irradiation, as discussed in Section 3.3.3. One possible factor is the increase in crosslinking density caused by additional crosslinking reactions activated by microwave energy, which gives rise to a denser polymer network requiring more energy for thermal decomposition. Another contributing factor may be the improvement in interfacial adhesion between the filler particles and the polymer matrix due to localized heating at the interface, which can reduce the formation of microvoids and defects that serve as pathways for volatile degradation products. In addition, microwave treatment may promote molecular relaxation and partial relief of internal stresses, resulting in a more equilibrated structure with fewer weak points vulnerable to thermal attack [57,62,64,90].

The thermal stability index provides a comprehensive measure of overall thermal performance by integrating information about both the onset and progression of decomposition. The THRI values increase progressively from 150.6 for the neat UPR to 159.6 for the untreated filled composite and further to 165.8 for the MW-post-cured composite. This progressive increase confirms the beneficial effects of both filler incorporation and microwave post-treatment on the thermal stability of the composites.

The residual mass at 800 °C provides clear evidence of the inorganic filler content. The neat UPR leaves only 3.08 wt.% residue, corresponding to carbonaceous char and inorganic impurities. In contrast, the untreated filled composite retains 24.32 wt.% of its initial mass at 800 °C, which closely corresponds to the original brick dust content. Notably, the MW-post-cured composite exhibits a slightly higher residue of 27.45 wt.%, which may indicate that microwave treatment promotes additional carbonization or more complete retention of organic components strongly bound to the filler surface [2,5]. This observation, combined with the unchanged filler content, further supports the conclusion that microwave treatment modifies the polymer matrix structure rather than altering the inorganic phase.

The Vicat softening temperature (T_v_), which characterizes the heat resistance of the materials under load, follows a similar trend. The neat UPR exhibits T_v_ = 140 °C, which increases to 160 °C for the untreated filled composite (a 20 °C improvement) and further to 171 °C for the MW-post-cured composite (a 31 °C improvement relative to the neat resin and an 11 °C improvement relative to the untreated filled composite). This progressive increase in heat resistance correlates well with the TGA results and confirms that both filler incorporation and microwave post-curing enhance the thermal stability of the composites. The improvement in T_v_ can be attributed to the restricted mobility of polymer chains in the presence of filler particles and the increased crosslinking density achieved through microwave post-treatment [19,56].

The 31 °C increase in Vicat softening temperature (from 140 °C to 171 °C) is of practical industrial significance, expanding the service temperature range of the material. This improvement makes the composite suitable for automotive under-hood components, electrical housings, and construction panels, where retention of mechanical properties under moderate thermal loads is required. While not intended for high-temperature applications beyond 200 °C, the material offers a competitive balance of thermal and mechanical performance for moderate-temperature applications.

The thermal stability improvements correlate well with the mechanical property enhancements reported in Section 3.3. The composite exhibiting the highest thermal stability parameters for the MW-post-cured sample also demonstrated the best overall mechanical performance. This correlation supports the conclusion that microwave post-curing simultaneously enhances multiple aspects of composite performance through the formation of a denser, more perfect polymer network with improved matrix–filler interfacial adhesion. Thus, the thermal analysis confirms that the optimal composite formulation (100 UPR: 50 BD) subjected to microwave post-curing for 120 s possesses not only superior mechanical properties but also enhanced thermal stability, making it suitable for applications requiring combined mechanical and thermal performance.

### 3.6. Water Absorption Behavior

**Figure 13:**
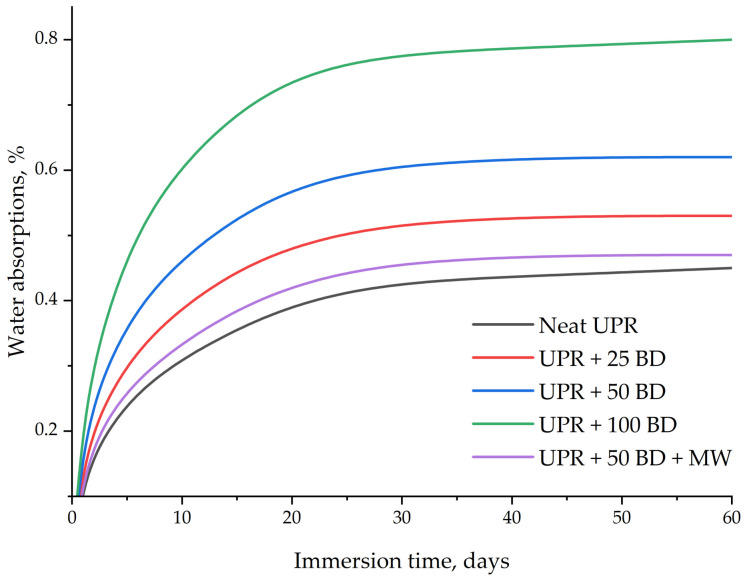
Water absorption kinetics of UPR/BD composites at 23 °C.

The water absorption characteristics of polymer composites are critical parameters for applications in humid environments, as moisture ingress can lead to plasticization, hydrolysis, and degradation of mechanical properties [20,91]. In this study, the water absorption behavior of UPR/BD composites was investigated according to the procedure described in Section 2.3.8. Figure 13 presents the water absorption kinetics for all investigated compositions, and Table 8 summarizes the equilibrium water absorption values.

The incorporation of brick dust significantly influences the water absorption behavior of the composites. The equilibrium water content increases progressively with filler loading: from 0.45% for the neat UPR to 0.53% at 25 phr BD, 0.62% at 50 phr BD, and dramatically to 0.80% at 100 phr BD. This increase can be attributed to several factors. First, brick dust possesses a hydrophilic nature, as revealed by XRF and FTIR analysis (Section 3.1), containing aluminosilicate minerals with surface hydroxyl groups that readily adsorb water molecules through hydrogen bonding. Second, at higher filler loadings, incomplete wetting of particles by the resin can lead to the formation of microvoids at the matrix–filler interface, which serve as preferential pathways for water ingress [92,93]. Third, at 100 phr BD, particle agglomeration creates capillary channels that facilitate water transport through the material, explaining the dramatic increase in equilibrium absorption. Finally, the presence of filler particles may disrupt polymer chain packing, increasing the free volume available for water molecules.

Remarkably, microwave post-curing of the composite containing 50 phr BD reduced the equilibrium water absorption to 0.47%, which is only 4.4% higher than that of neat UPR and substantially lower than that of the untreated composite (0.62%). This improvement can be attributed to the microstructural changes induced by microwave treatment. First, as confirmed by gel fraction measurements (Section 3.3.4), microwave post-curing increased the crosslinking density from 95.0% to 97.8%. The resulting denser polymer network reduced the free volume available for water molecules and restricted their mobility. Second, improved adhesion between the filler particles and the matrix likely reduced the number of interfacial microvoids that can act as water reservoirs and diffusion pathways. Third, microwave-induced relaxation of internal stresses may have produced a more equilibrated structure with fewer microdefects capable of initiating water uptake.

To assess the practical implications of water absorption, the mechanical properties of selected samples were evaluated after saturation (60 days immersion) and after re-drying (24 h at 60 °C). The property retention and reversibility index were calculated according to the procedures described in Section 2.3.8. The results are presented in Table 9.

The partial recovery of mechanical properties upon re-drying indicates that both reversible (plasticization) and irreversible (hydrolysis, microcracking) degradation mechanisms occur. The composite with 100 phr BD shows the poorest property retention after saturation (74.4% for flexural strength) and the lowest recovery upon re-drying (87.2%), indicating significant irreversible damage due to interfacial degradation. In contrast, the MW-post-cured composite exhibits exceptional retention of properties after saturation (92.7% for flexural strength) and nearly complete recovery after re-drying (98.2%), confirming that the improved interfacial adhesion and higher crosslinking density confer superior resistance to moisture-induced degradation. The reversibility index provides a measure of the reversible component of degradation: the neat UPR shows 77.8% reversibility, UPR + 50 BD shows 70.8%, UPR + 100 BD shows only 50.0%, while the MW-treated sample exhibits the highest reversibility at 87.5%. This indicates that degradation in the microwave-treated composite is predominantly due to reversible plasticization rather than irreversible interfacial damage.

These results confirm that microwave post-curing not only enhances the initial mechanical properties but also significantly improves the durability of UPR/BD composites in humid environments, expanding their potential for applications requiring long-term moisture resistance.

### 3.7. Mechanisms of Structural Modification: Comparison Between Microwave Irradiation and Advanced Chemical Functionalization Strategies

In modern polymer materials science, enhancing the performance characteristics of thermosetting resins traditionally relies on a combination of physical reinforcement and complex chemical modification. As systematized in a recent review [94,95,96,97], the chemical engineering of phase boundaries and matrix structures serves as a key strategy to overcome filler agglomeration and the inherent brittleness of highly cross-linked polymers. An analysis of recent advances in the field of UPRs reveals that functional group grafting techniques are actively employed for these purposes; examples include the incorporation of epoxy-functionalized nano-silica [72], the utilization of hyperbranched polyesters [98] and polysiloxanes [99], as well as the surface modification of 2D nanomaterials such as graphene oxide (including functionalization with ionic liquids and magnetic nanoparticles) [100,101] and hexagonal boron nitride [99]. The structural reinforcement mechanisms observed in the present study upon microwave treatment of UPR-BD composites exhibit profound physicochemical parallels with the effects of these advanced chemical functionalization methods, effectively acting as their highly efficient electrophysical counterpart.

The primary objective of chemical filler modification (e.g., the use of ionic liquids for graphene oxide [101] or the modification of boron nitride with sucrose and amino groups [99]) is to establish chemical affinity between the polar surface of inorganic particles and the hydrophobic polymer matrix, thereby preventing phase separation and ensuring homogeneous dispersion. In our study, a similar structural coupling effect is achieved via dipole polarization within the microwave field. Intensive localized heating induces deep desorption of physically bound moisture from the micropores of the aluminosilicate brick dust particles, exposing the surface hydroxyl groups. Concurrently, a brief yet sharp drop in oligomer viscosity within the microwave field ensures ideal wetting of the filler at the submicron level. Similar to chemically cross-linked nanocomposites [72,99], where a strong interfacial bond shifts the failure mechanism from adhesive to cohesive, SEM analysis of the MW-treated specimens (Figure 11) reveals complete encapsulation of the particles and the formation of ultrathin polymer veils. This provides direct evidence that the interfacial adhesion strength exceeds the cohesive strength of the polymer matrix itself.

A traditional challenge for thermosetting plastics is the trade-off between stiffness and toughness (fracture resistance). To address this, hyperbranched polymers are actively employed [98,99]; these integrate into the UPR network, forming flexible yet strong cross-link junctions capable of efficiently dissipating the energy of a propagating crack via localized deformations (crack deflection). Electrophysical modification enables a similar synergistic effect without the synthesis of complex macromolecules. The microwave field initiates localized “hot spots” in the vicinity of the filler particles, accelerating the polymerization of residual unsaturated groups throughout the bulk volume, as evidenced by an increase in the gel fraction content up to 97.8%. Simultaneously, bulk microwave heating promotes deep relaxation of internal thermal stresses. This results in the formation of a more perfect matrix capable of plastic deformation prior to failure (as indicated by the development of ragged edges on the fracture surfaces). This specific mechanism of structural relaxation and post-curing is responsible for the anomalous 119% increase in impact strength while maintaining or even increasing the flexural modulus (a 49.7% growth).

In chemically modified systems, the functionalization of 2D particles (GO, BN) promotes their aligned orientation (e.g., under a magnetic field [100]), which creates a complex tortuous path that blocks the permeation of water molecules, heat, and oxygen [100,101]. In the investigated UPR/BD composites, microwave processing leads to a similar restriction of the free volume. The elimination of microvoids at the phase interface and the densification of the three-dimensional polymer network eliminate the capillary channels responsible for moisture diffusion. Consequently, the equilibrium water absorption of the composite filled with 50 phr of brick dust decreases from 0.62% to 0.47% (approaching the values of the neat resin), and the property reversibility index reaches 87.5%, effectively suppressing the irreversible hydrolytic degradation of the interfacial layer.

Thus, the comparative analysis demonstrates that microwave post-curing serves as a powerful, comprehensive tool for structural engineering. The physicochemical effects induced by it (enhanced wettability, network densification, stress relaxation, and particle encapsulation) are functionally identical to the outcomes of complex chemical functionalization and the application of hyperbranched polymers. However, the proposed electrophysical approach circumvents multi-step chemical synthesis, the use of volatile solvents, and expensive coupling agents, offering a highly processable and sustainable pathway for fabricating highly loaded composites from secondary mineral resources.

## 4. Conclusions

This study demonstrated an effective approach for the valorization of technogenic BD as a reinforcing filler for UPR composites, combined with MW treatment at different stages of material fabrication. Comprehensive characterization confirmed that brick dust is an environmentally safe aluminosilicate material with a mean particle size of 3–6 μm, plate-like morphology, and surface hydroxyl groups favorable for interfacial interaction with the polymer matrix.

Optimization of filler content revealed that the composite with 50 phr BD exhibited the best balance of mechanical properties, including a flexural strength of 82 MPa (+6.5%), flexural modulus of 5510 MPa (+134%), tensile strength of 42 MPa (+11%), tensile modulus of 2875 MPa (+99%), impact strength of 6.3 kJ/m^2^ (+40%), and Shore D hardness increased from 78 to 83. Among the three microwave treatment strategies investigated, post-curing of the fully polymerized composite for 120 s proved most effective, yielding the highest improvements: flexural strength of 110 MPa (+34.1%), flexural modulus of 8250 MPa (+49.7%), tensile strength of 56 MPa (+33.3%), impact strength of 13.8 kJ/m^2^ (+119%), Shore D hardness of 88, and gel fraction content of 97.8%.

Morphological analysis revealed that the MW post-cured composite exhibited a tortuous fracture surface with ultra-thin polymeric veils replicating the scaly filler structure and jagged tear edges, indicating cohesive failure through the matrix rather than interfacial debonding, which provides direct visual evidence for the high energy dissipation capacity of this material.

Thermal stability analysis showed progressive improvements, with T5% increasing from 262 °C (neat UPR) to 286 °C (UPR + 50 BD) and further to 308 °C (MW post-cured), while the THRI rose from 150.6 to 165.8 and the Vicat softening temperature increased from 140 °C to 171 °C.

Water absorption studies demonstrated that MW post-curing reduced equilibrium water uptake from 0.62% to 0.47% (approaching the neat UPR value of 0.45%) and achieved a reversibility index of 87.5%, with the MW-treated composite retaining 92.7% of its flexural strength after saturation and recovering 98.2% upon re-drying.

Overall, the combination of 50 phr brick dust with subsequent microwave post-curing (120 s) yields a composite with superior mechanical performance, enhanced thermal stability, and improved durability against moisture ingress, offering a sustainable route for utilizing ceramic waste as an active filler while simultaneously upgrading the polymer matrix structure through electrophysical modification, making this material promising for applications requiring high impact resistance, good thermal stability, and long-term reliability in humid environments.

## Figures and Tables

**Table 1:** Technical specifications of Aropol™ M105TB unsaturated polyester resin.

Property	Value
Brookfield viscosity (RV2), mPa·s	1400
Cone & Plate viscosity, mPa·s	180
Styrene content, %	~37
Density, kg/dm^3^	1.1
Gel time with 1% MEKP-50, min	40
Exothermic peak, °C	110

**Table 2:** Technical specifications of BUTANOX^®^ M-50 (MEKP) initiator.

Property	Value
Active oxygen content, %	8.8–9.0
Density at 20 °C, g/cm^3^	1.180
Viscosity at 20 °C, mPa·s	24
Water content, %	max. 3

**Table 3:** Mechanical properties of polyester composites modified with brick dust.

Composition (phr)	σ_f_, MPa	E_f_, MPa	σ_t_, MPa	E_t_, MPa	a_i_, kJ/m^2^	Shore D Hardness
100 UPR	77 ± 3.1	2355 ± 95	38 ± 1.8	1444 ± 60	4.5 ± 0.2	78 ± 2
100 UPR + 25 BD	79 ± 3.2	3350 ± 125	40 ± 1.9	2105 ± 90	4.8 ± 0.2	80 ± 2
100 UPR + 50 BD	82 ± 3.3	5510 ± 215	42 ± 2.0	2875 ± 116	6.3 ± 0.3	83 ± 2
100 UPR + 75 BD	80 ± 3.2	6565 ± 252	40 ± 1.9	3265 ± 125	4.6 ± 0.2	84 ± 2
100 UPR + 100 BD	78 ± 3.1	9455 ± 390	38 ± 1.8	4435 ± 176	3.4 ± 0.2	85 ± 2

Note: σ_f_—flexural strength; E_f_—flexural modulus; σ_t_—tensile strength; E_t_—tensile modulus; a_i_—impact strength.

**Table 4:** Mechanical properties of polyester composites modified with brick dust under different microwave treatment conditions.

MW Treatment Stage	Duration, s	σ_f_, MPa	E_f_, MPa	σ_t_, MPa	E_t_, MPa	a_i_, kJ/m^2^	Shore D Hardness
-	-	82 ± 3.3	5510 ± 215	42 ± 2.0	2875 ± 116	6.3 ± 0.3	83 ± 2
Brick dust (Filer)	30	85 ± 3.4	5785 ± 231	44 ± 2.0	3010 ± 121	7.5 ± 0.5	84 ± 2
	60	103 ± 4.1	7198 ± 281	57 ± 2.3	3833 ± 148	11.5 ± 0.6	86 ± 2
	90	88 ± 3.5	6845 ± 271	52 ± 2.1	3461 ± 138	11.9 ± 0.7	85 ± 2
UPR with BD (Oligomer)	2	84 ± 3.3	5710 ± 225	44 ± 2.0	2985 ± 119	6.8 ± 0.4	84 ± 2
	3	90 ± 3.6	7625 ± 302	51 ± 2.1	3350 ± 125	10.5 ± 0.5	85 ± 2
	5	88 ± 3.5	7515 ± 299	48 ± 2.0	3210 ± 121	9.6 ± 0.5	85 ± 2
Fully polymerized composite	60	85 ± 3.5	6080 ± 245	45 ± 1.9	2980 ± 115	9.5 ± 0.5	84 ± 2
	120	110 ± 4.1	8250 ± 315	56 ± 2.3	3410 ± 129	13.8 ± 0.7	88 ± 2
	180	103 ± 4.2	7955 ± 300	47 ± 2.0	3178 ± 127	11.1 ± 0.6	86 ± 2

Note: σ_f_—flexural strength; E_f_—flexural modulus; σ_t_—tensile strength; E_t_—tensile modulus; a_i_—impact strength.

**Table 5:** Comparative efficiency of microwave treatment strategies applied at different stages of UPR/BD composite fabrication.

Property	Filler Pretreatment	Oligomer Modification	Composite Post-Curing
Flexural strength	+25.6%	+9.8%	+34.1%
Flexural modulus	+30.6%	+38.4%	+49.7%
Tensile strength	+35.7%	+21.4%	+33.3%
Tensile modulus	+33.3%	+16.5%	+18.6%
Impact strength	+82.5%	+66.7%	+119.0%

**Table 6:** Gel fraction content of UPR/BD composites (50 phr filler) under different microwave treatment conditions.

Treatment Mode	Microwave Exposure Time (s)	Gel Fraction Content, %
Without MW treatment (control)	–	95.0 ± 0.8
MW treatment of brick dust	60	95.8 ± 0.8
MW modification of oligomer	3	96.5 ± 0.7
MW post-curing of composite	120	97.8 ± 0.6

**Table 7:** Thermal stability data of UPR/BD compositions.

Samples	T_5%_, °C	T_10%_, °C	T_30%_, °C	T_50%_, °C	Residues at 800 °C, wt.%	THRI	T_v_
Neat UPR	262	300	337	365	3.08	150.6	140
UPR + 50 BD	286	315	352	384	24.32	159.6	160
UPR + 50 BD + MW Post-cure	308	335	360	390	27.45	165.8	171

Note: Tv—Vicat heat resistance, THRI—thermal stability index.

**Table 8:** Equilibrium water absorption of UPR/BD composites.

Sample	Equilibrium Water Absorption, $W_{\infty }$ (%)	Change Relative to Neat UPR
Neat UPR	0.45	–
UPR + 25 BD	0.53	+17.8%
UPR + 50 BD	0.62	+37.8%
UPR + 100 BD	0.80	+77.8%
UPR + 50 BD + MW	0.47	+4.4%

**Table 9:** Mechanical properties of UPR/BD composites before and after water immersion.

Sample	Condition	Flexural Strength, MPa	Retention, %	Flexural Modulus, MPa	Retention, %
Neat UPR	Dry	77	–	2355	–
	Saturated	68	88.3	2100	89.2
	Re-dried	75	97.4	2280	96.8
UPR + 50 BD	Dry	82	–	5510	–
	Saturated	70	85.4	4680	85.0
	Re-dried	78	95.1	5250	95.3
UPR + 100 BD	Dry	78	–	9455	–
	Saturated	58	74.4	7100	75.1
	Re-dried	68	87.2	8200	86.7
UPR + 50 BD + MW	Dry	110	–	8250	–
	Saturated	102	92.7	7700	93.3
	Re-dried	108	98.2	8090	98.1

## Data Availability

The original contributions presented in this study are included in the article. Further inquiries can be directed to the corresponding author.
